# iCare Technique of Dissolving Ellanse M Nodules Using Collagenase: A Case Series and Experimental Study

**DOI:** 10.1111/jocd.70201

**Published:** 2025-04-28

**Authors:** Larry Wu

**Affiliations:** ^1^ iCare Medical Centre Singapore Singapore

**Keywords:** collagenase mixture, delay onset nodules, Ellanse, hyaluronidase

## Abstract

**Background:**

Ellanse is a polycaprolactone‐based collagen stimulator that is rarely associated with the formation of palpable nodules. The current management of palpable nodules include triamcinolone injection, consumption of oral methotrexate, and surgical excision. The absence of an effective reversal agent that is safe has limited the popularity of Ellanse as a facial rejuvenation modality.

**Objective:**

This article presents a dual focus (1) a case series demonstrating the efficacy of the iCare technique in treating 10 nodules from Ellanse M treatment in three patients and (2) an experimental study investigating the in vitro and in vivo dissolution of Ellanse M aliquots. In the case series, nodules, which have persisted for an average of 2 years following initial Ellanse M treatment, are effectively managed with the iCare technique. The iCare technique of dissolution involves injecting a collagenase mixture that is five times the initial injected volume of Ellanse. The case series finding demonstrates the iCare technique as a viable solution in managing Ellanse‐associated nodules. An allergy test is also conducted prior to injection of collagenase mixture to demonstrate the safety of collagenase mixture.

**Experimental Study:**

An experiment is conducted by mixing 0.1 mL Ellanse M aliquots with 0.5 mL collagenase mixture (Slide X). Controls are provided by adding 0.5 mL of lignocaine 2% and adrenaline 1:80 000 (Slide L), 0.5 mL hyaluronidase (Slide H), 0.5 mL of 40 mg/mL of triamcinolone (Slide T) separately to 0.1 mL Ellanse M aliquots.

**Results:**

Ellanse M is converted from a gel into a solution by collagenase mixture (X) while it is not affected by lignocaine and adrenaline (L), hyaluronidase (H), or triamcinolone (T). The conversion of Ellanse gel into a solution occurs within 5 min of adding the mixture.

**Conclusion:**

The case series demonstrates the iCare technique's efficacy in addressing delayed onset nodules associated with Ellanse treatment. The experimental study demonstrates in vitro and in vivo dissolution of injected Ellanse aliquots. This article offers a promising solution to significant issues associated with Ellanse treatment—namely, the formation of nodules and the lack of an effective fast‐acting reversal agent.

## 1 Introduction

Ellanse, distributed by Sinclair Pharmaceutical, is a polycaprolactone‐based collagen stimulator designed for patients seeking natural aesthetic results through neocollagenesis. It consists of 30% polycaprolactone microspheres suspended in 70% carboxymethylcellulose gel. Ellanse has two different preparations—Ellanse S which has a product longevity of 12 to 18 months and Ellanse M which has a longevity of 24 months [1]. Ellanse stimulates type 1 collagen production for facial [2] and hand rejuvenation [3]. Neocollagenesis has been demonstrated by the presence of new collagen formation around polycaprolactone microspheres [4].

Currently, the lack of a hyaluronidase equivalent for Ellanse has impeded the widespread adoption of Ellanse for non‐surgical facial rejuvenation. The iCare technique involves injecting a collagenase mixture that is five times the initiated estimated volume of Ellanse injected. Collagenase is a class of therapeutic enzymes involved in the cleavage of peptide bonds found in collagen [5] and they may represent a novel modality in the management of Ellanse gel and associated nodules. The following case series of 3 patients illustrates the successful use of iCare technique in the dissolution of delayed onset nodules (DON) associated with Ellanse treatment.

## 2 Case Series

### 2.1 Method

The case series of 3 patients consists of 2 existing patients and one external referral. The main complaints are palpable nodules that are present more than 2 years following Ellanse M treatment. The size of nodules is measured both before and after collagenase mixture treatment. Informed consent was obtained from all patients and the research was conducted in accordance with the principles outlined in the Declaration of Helsinki, 2018 [6]. In addition, ethics approval for this study was granted by the ethics review board (Approval: IMAC/2024/733/004).

**FIGURE 1:**
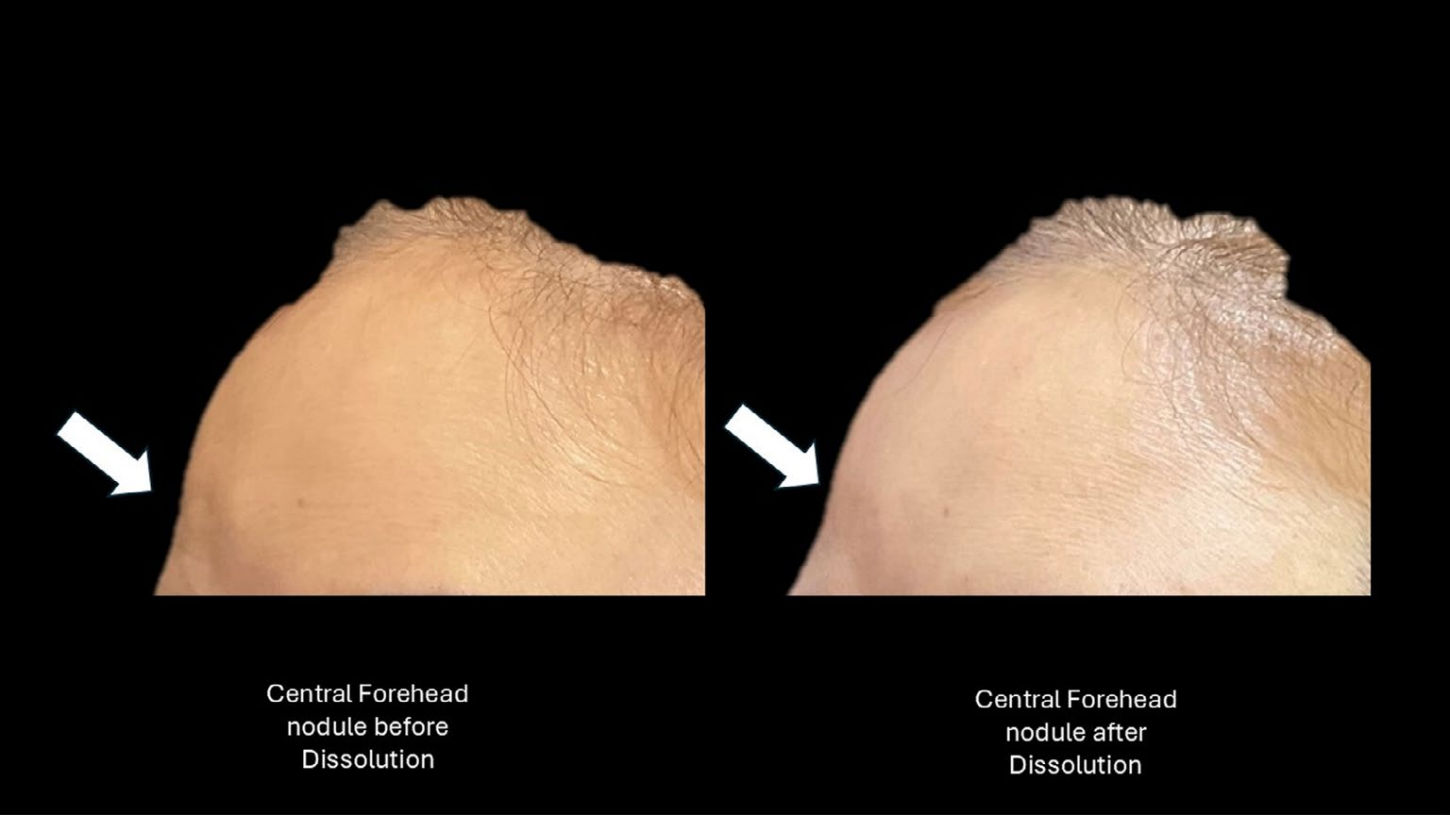
Demonstrating forehead nodule subsiding 2 days after treatment with collagenase mixture.

Patient 1 is a 36‐year‐old female who received forehead rejuvenation with 2 syringes of Ellanse M 2 years ago. She has presented for further facial treatment with Ellanse, however she has reported a small 5 mm nodule over her forehead. On palpation, the lump is mobile and is probably related to superficial treatment with Ellanse. The iCare technique was performed using a 31 G insulin needle (BD Ultrafine II) to inject 0.5 mL of collagenase mixture and the nodule was impalpable after 5 min of massage. She proceeds to receive further Ellanse treatment to her temporal regions, mid‐cheek, and nasolabial folds. A review of the patient 2 days later demonstrates the forehead nodule has been reduced to a small palpable lump of less than 1 mm (see Figure 1 below).

Patient 2 is a 60‐year‐old female who received Ellanse treatment 2 years ago. She complained of 8 palpable nodules over the forehead, temporal region, orbital rim as well as pre‐jowl sulcus. The nodules range from 10 mm to 25 mm in diameter. The largest nodule is over the central forehead region measuring 25 mm. Intralesional triamcinolone 0.2 mL (40 mg/mL) 1 month ago has partially reduced the size of the nodule. The iCare technique is performed by intralesional injection of nodules over the forehead, orbital rim, pre‐jowl sulcus, and temporal nodules with a collagenase mixture. The forehead nodule reduced within 10 min of injection to a 10 mm nodule. The remaining nodules have experienced a 50% reduction in size. The forehead nodule is injected again 1 month later with successful resolution of the nodule.

**FIGURE 2:**
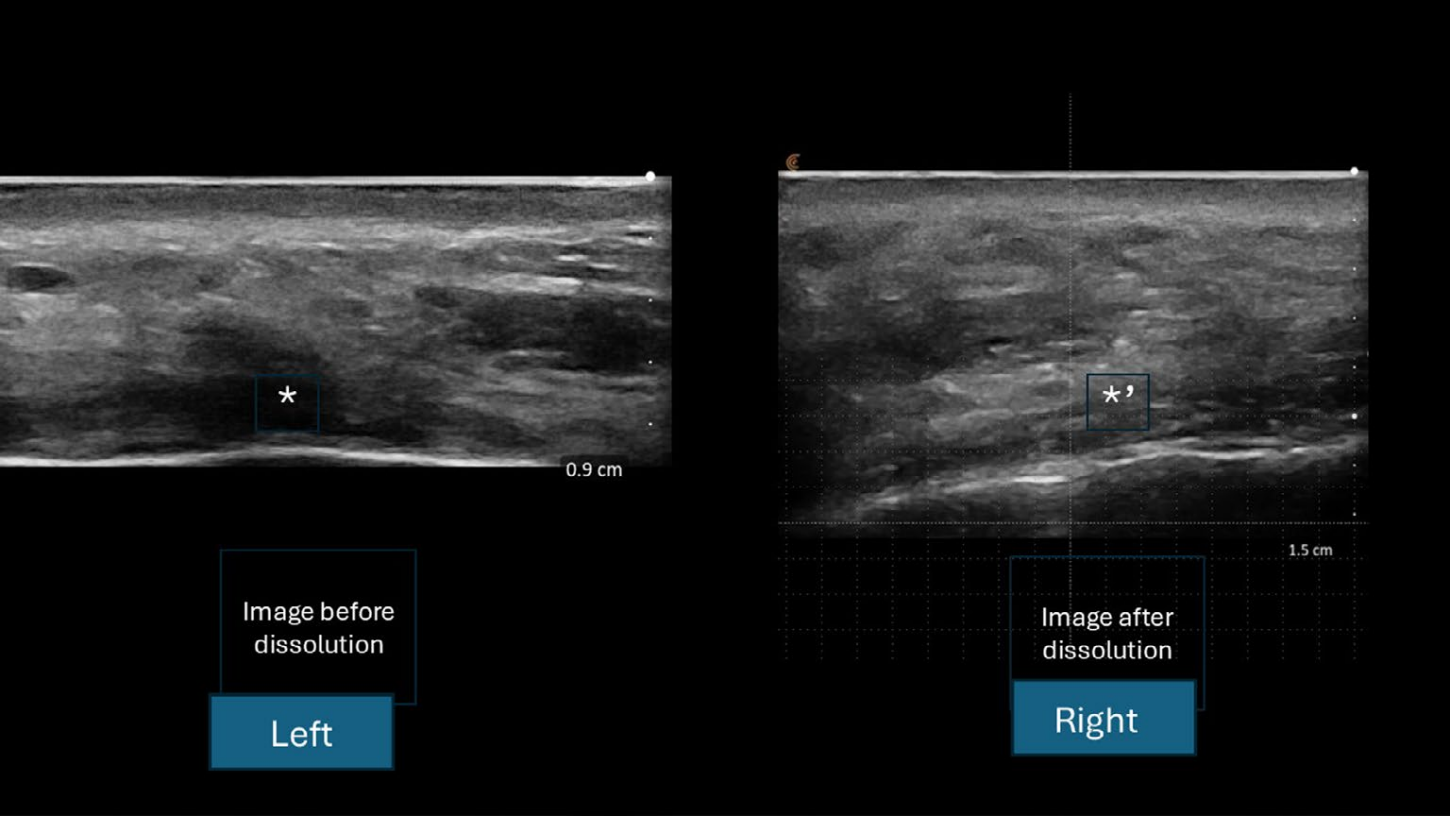
Left—Single star * demonstrates hypoechoic nodule in the chin. Right—*′ demonstrate hypoechoic nodule has resolved.

Patient 3 is a 48‐year‐old male who had 0.2 mL Ellanse M injected over the left chin mentum 2 years ago. An ultrasound scan reviews a dark hypoechoic shadow over the chin associated with a reduction in posterior acoustic shadowing consistent with previous Ellanse treatment (see Figure 2).

**FIGURE 3:**
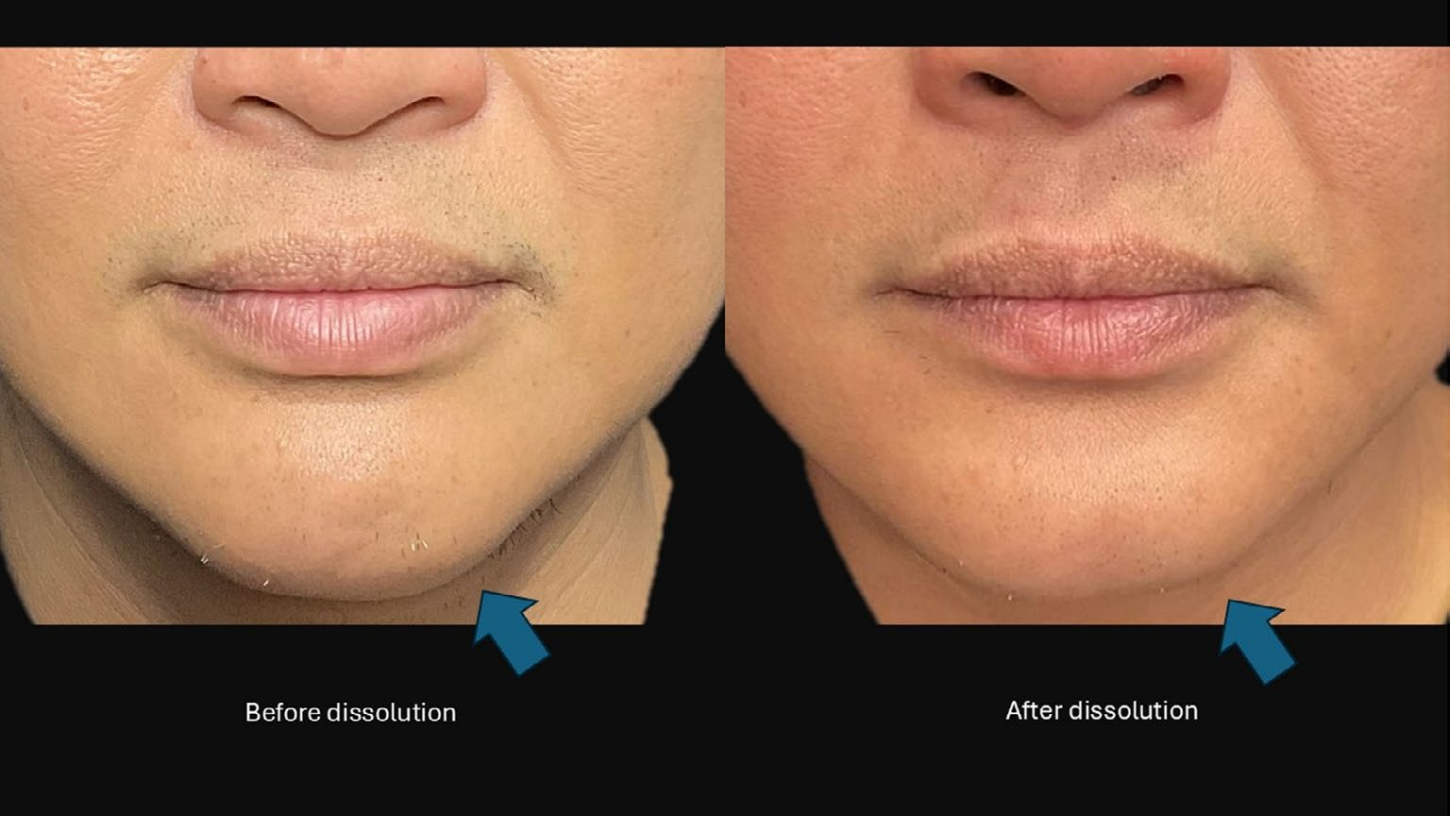
Left chin nodule has been treated with collagenase mixture (Blue arrow) and the treated area is depressed, demonstrating effective resolution.

An injection of 1 mL of collagense mixture has resulted in the complete dissolution of collagen formation. A repeat scan the following day demonstrates the hypoechoic shadow associated with Ellanse has disappeared (see Figure 2). The left chin mentum that is treated with collagenase mixture subsequently has a depressed appearance (see Figure 3).

## 3 Experimental Study

A 3‐part (Part 1, 2, and 3) experimental study is also conducted to demonstrate the in vitro, in vivo dissolution of Ellanse gel as well as the safety of collagenase mixture.

**FIGURE 4:**
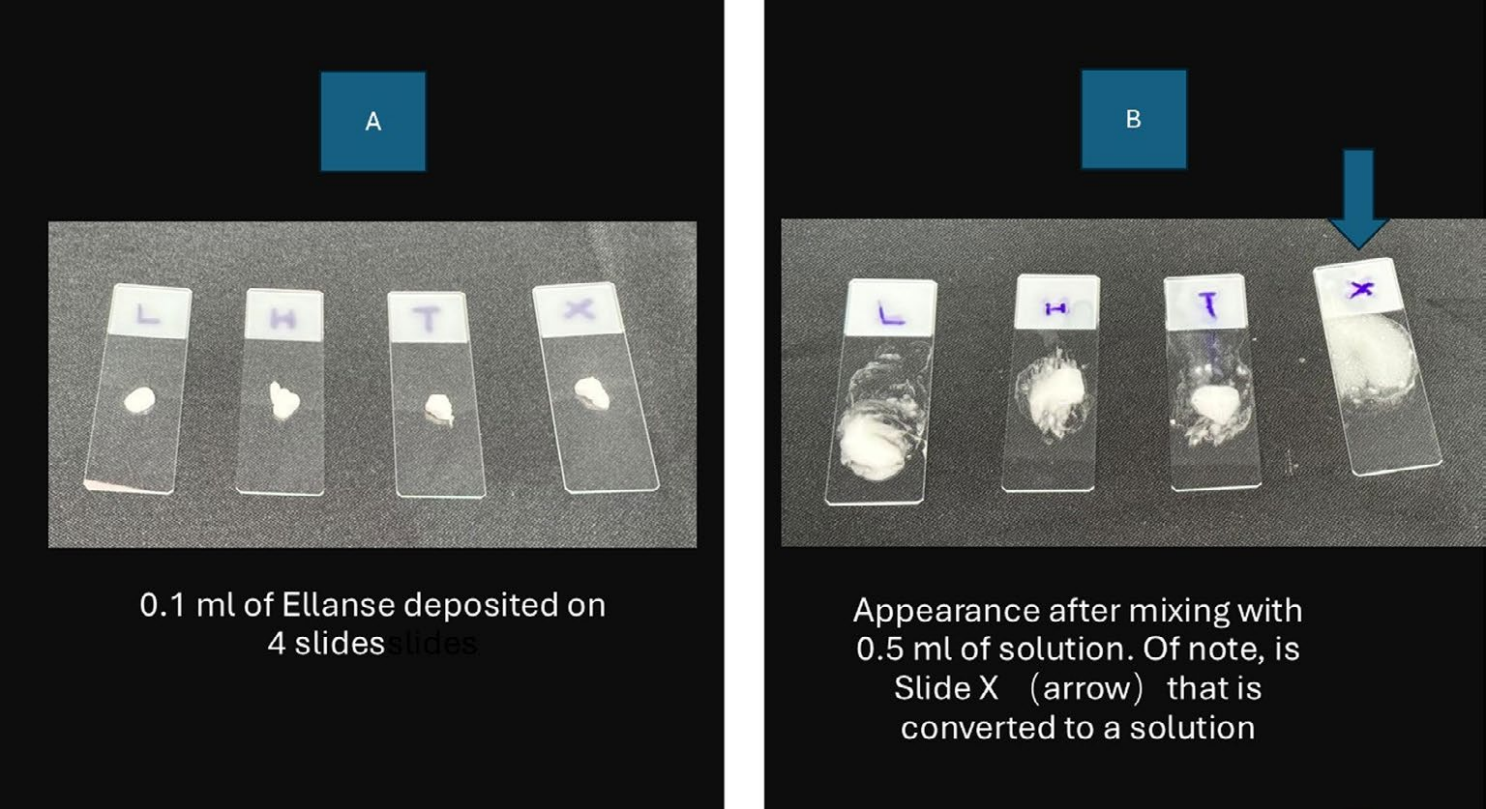
(A) 0.1 mL of Ellanse M deposited on Slide L, Slide H, Slide T, and Slide X. (B) After treatment. Ellanse M on slide X has liquefy into a solution.

Part 1: In vitro dissolution of Ellanse M aliquots with collagenase mixture (X) with lignocaine and adrenaline, triamcinolone as well as hyaluronidase serving as controls (Figure 4).

**FIGURE 5:**
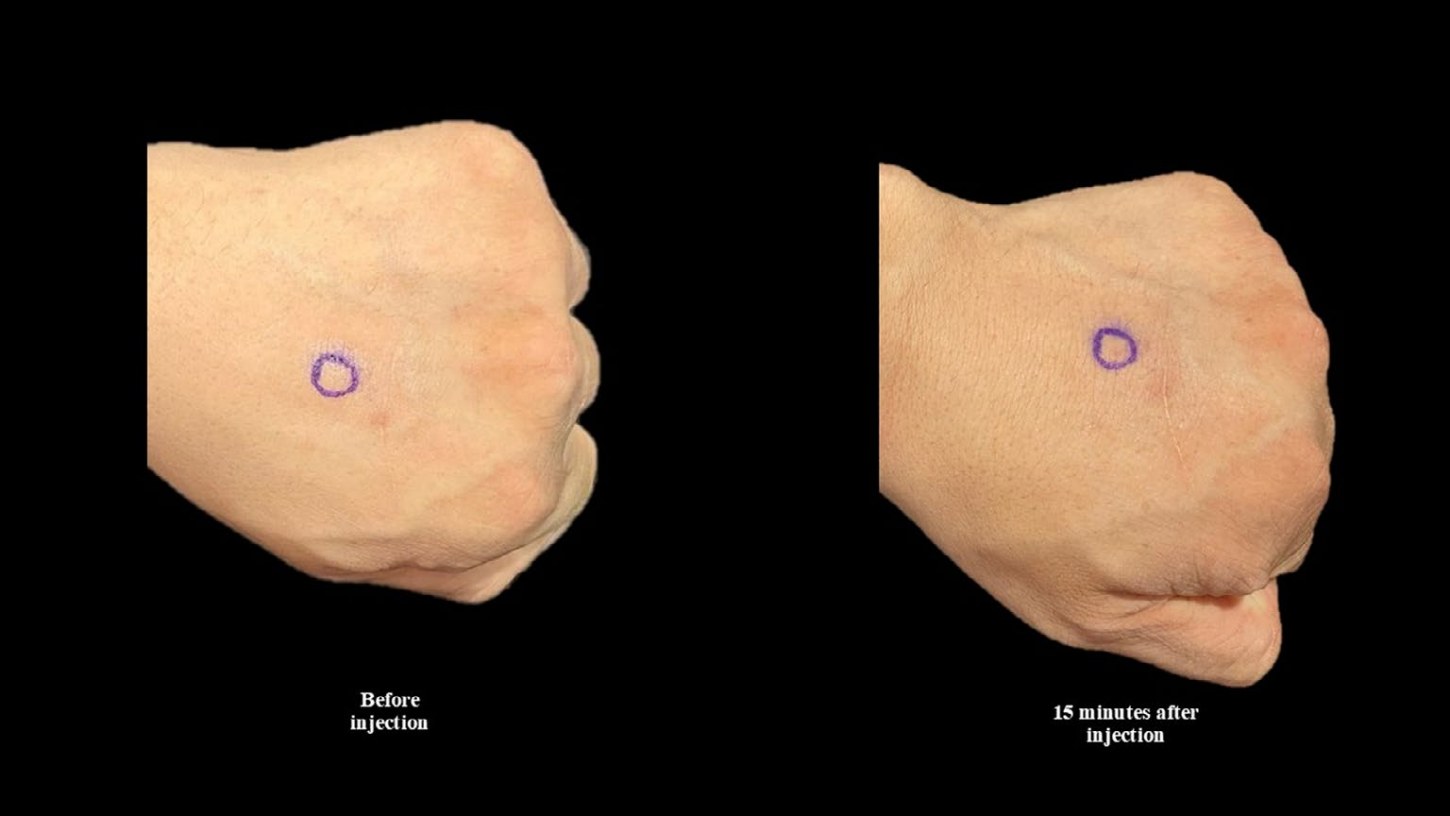
The dorsum of the hand before (left) and 15 min after 1 mL of collagenase mixture (right) is injected in the blue circle. There is no evidence of inflammation suggesting the absence of allergy to collagenase mixture.

Part 2: Allergy test of collagenase mixture (Figure 5).

**FIGURE 6:**
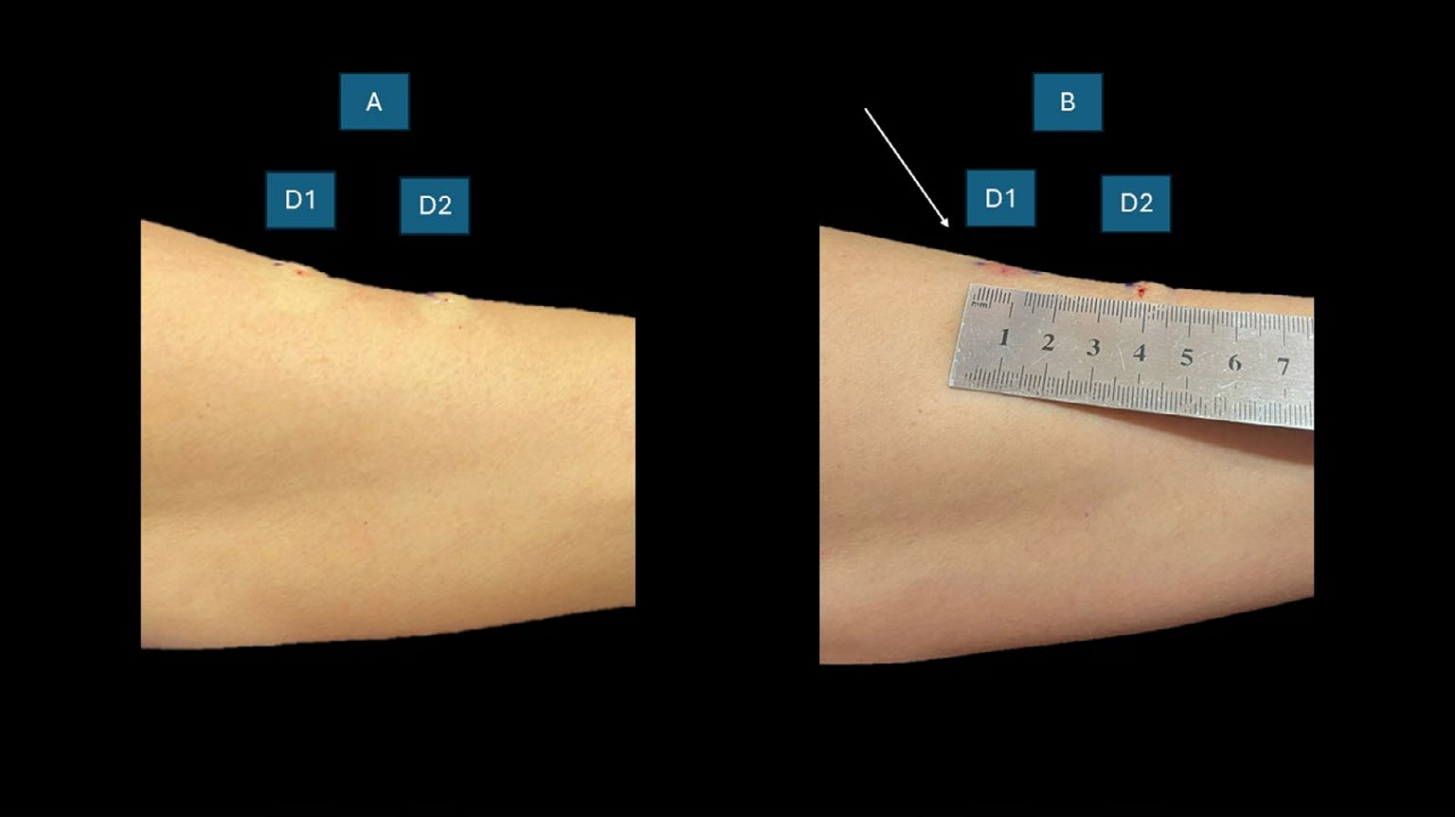
(A) Demonstrates 2 × 0.1 mL (D1 and D2) intradermal deposits of Ellanse M. (B) Demonstrates dissolution (flattening) of Ellanse M (D1) after 0.5 mL of collagenase mixture and 5 min of massage (see white arrow).

Part 3: In vivo dissolution of intradermal deposits to Ellanse M (Figure 6).

**Part 1**

In vitro dissolution of Ellanse M: 0.1 mL droplet of Ellanse M is mixed for 5 min with the following mixtures

1. 0.5 mL of Lignocaine 2% and adrenaline 1:80 000 (Slide L).
2. 0.5 mL of Hyaluronidase (1500 IU in 1 mL) (Slide H).
3. 0.5 mL of Triamcinolone 40 mg/mL (Slide T).
4. 0.5 mL of Collagenase mixture (Slide X).

### 3.1 Results

Following the introduction of collagenase mixture, the rheology of Ellanse, that is, G′ and viscosity is disrupted and the Ellanse is converted from a gel to a solution within 5 min of the mixture. Lignocaine and adrenaline (Slide L), triamcinolone (Slide T), as well as hyaluronidase (Slide H), did not affect the Ellanse gel's viscosity (see Figure 4).

1. Slide L: 0.1 mL Ellanse M aliquot +0.5 mL Lignocaine 2% and Adrenaline 1:80 000 demonstrating the gel structure of Ellanse is intact 5 min after mixture.
2. Slide H: 0.1 mL Ellanse M aliquot +0.5 mL of hyaluronidase demonstrating the gel structure of Ellanse is intact 5 min after mixture.
3. Slide T: 0.1 mL of Ellanse M aliquot +0.5 mL of triamcinolone 40 mg/mL.
4. Slide X: 0.1 mL of Ellanse M aliquot +0.5 mL of collagenase mixture demonstrating the gel is converted in a solution (Blue arrow) (see Video 1).

**VIDEO 1:** Demonstrating that Ellanse gel is converted to a solution and is sliding off the microscopic slide 5 min after the collagenase mixture. Video content can be viewed at https://onlinelibrary.wiley.com/doi/10.1111/jocd.70201

**Part: Allergy testing**

Allergy testing is performed to demonstrate the safety of collagenase mixture on the author's hand (see Figure 5).

## 4 Conclusion

The purpose of this case series and 3‐part experiment serves dual objectives: 1. to describe the iCare technique of using collagenase mixture in addressing nodule formation secondary to Ellanse 2. to demonstrate the efficacy of collagenase mixture in dissolving newly injected Ellanse. One of the main challenges of Ellanse treatment is the inadvertent nodule formation with a quoted incidence of 0.023% [7]. The mechanism of nodule formation has been postulated to be secondary to excessive Ellanse injected, incorrect injection depth and injection in a highly mobile location such as muscles [8]. The absence of a fast‐acting, effective dissolution agent for Ellanse has hindered the widespread adoption of Ellanse as a facial rejuvenation modality. The current management techniques involving triamcinolone injections, consumption of oral methotrexate, and surgery have its own inherent disadvantages [9]. In addition, the successful resolution rate of nodules has been limited (9%) in one case series [10].

Our case series illustrates the use of iCare technique to address inadvertent nodule formation associated with Ellanse treatment in three patients. Based on our case series of 10 nodules in the three patients, for nodules up to 5 mm in diameter, injection of five times the estimated nodule volume of collagenase mixture is effective. In order to address lesions larger than 10 mm, more than one treatment session is recommended. The three patients did not experience any adverse events such as allergy, infection, or skin necrosis. The potential impact of this technique may represent a shift in the management algorithm for addressing Ellanse‐related nodules to a safer, less disfiguring method. This can greatly impact patient's satisfaction and increase the adoption of Ellanse treatment.

The result of the in vitro and in vivo experiment also demonstrates the efficacy of the collagenase mixture in dissolving newly injected Ellanse M within 5 min. The speed of dissolution of injected Ellanse represents a breakthrough in patient management. This rapid dissolution also represents a notable advancement in Ellanse training, providing reassurance to new injectors and facilitating better handling of misplaced Ellanse.

The limitation of this article is the limited sample size of three patients, but it is compensated by the presence of 10 nodules in these three patients. Additional studies are needed to assess if the nodules would recur following injection and the number of sessions required to effectively address larger nodules. Further studies are needed to evaluate the efficacy of collagenase mixture for inadvertent intravascular injection of Ellanse and to determine optimal dosing protocols.

## Disclosure

This study evaluates the iCare technique, which utilizes a collagenase mixture to dissolve Ellanse M nodules and associated collagen formations. potentially enhancing the safety and popularity of Ellanse as a facial rejuvenation modality.

## Conflicts of Interest

The author declares no conflicts of interest.

## Data Availability

The data that support the findings of this study are available on request from the corresponding author. The data are not publicly available due to privacy or ethical restrictions.
